# Ionic Liquid Electrolyte Suppresses Deep Sodiation in Nb_4_P_2_S_21_/Mo_2_CT_*x*_ Enabling Transition from Mixed-Voltage to Pure High-Voltage Operation for Sodium-Ion Battery Cathodes

**DOI:** 10.1021/acsami.5c10976

**Published:** 2025-10-01

**Authors:** Heng Li, Lei Zheng, Zhongquan Liao, Vlastimil Mazánek, Qiliang Wei, Tomáš Hartman, Saeed Ashtiani, Bing Wu, Zdenek Sofer

**Affiliations:** † Department of Inorganic Chemistry, 52735 University of Chemistry and Technology Prague, Technická 5, 166 28 Prague, Czech Republic; ‡ Fraunhofer Institute for Ceramic Technologies and Systems (IKTS), Maria-Reiche-Strasse 2, 01109 Dresden, Germany; § Institute of Micro/Nano Materials and Devices, 165069 Ningbo University of Technology, Ningbo 315211, P. R. China

**Keywords:** sulfur-equivalent cathode, sulfur-rich material, Nb_4_P_2_S_21_, MXene, ionic liquid electrolyte, sodium-ion battery

## Abstract

Elemental sulfur has garnered significant attention due to its low cost and high theoretical capacity; however, its reliance on ether electrolytes leads to the formation of soluble polysulfides, thereby limiting its application. Sulfur-rich transition metal polysulfides demonstrate potential as sulfur-equivalent cathodes to replace conventional sulfur in alkali metal–sulfur batteries; however, adequate research in this area remains unrevealed. In this study, we investigate the Nb_4_P_2_S_21_ in carbonate, ether, and ionic liquid electrolytes for sodium-ion battery testing. The material exhibits a high discharge capacity exceeding 1000 mAh/g and a prolonged discharge plateau at low potentials in both ether and carbonate electrolytes, same with other high-capacity phosphorus sulfide anodes via conversion reactions. When switching to the NaTFSI/[Emim]­TFSI ionic liquid electrolyte, 96.3% of the initial discharge capacity in the 0–3 V range is retained above 0.8 V, with the suppression of low-voltage redox activity. This shift is attributed to the cointercalation of Na^+^ and Emim^+^ ions, preventing the materials from deep sodiation at lower voltage range. The incorporation of Mo_2_CT_*x*_ MXene into the material further reduces electrochemical polarization and enhances cycle stability. During 100 cycles, a self-activation phenomenon occurs, resulting in a maximum capacity of 384 mAh/g, while the median voltage remains above 1.5 V, predominantly governed by a pair of reversible redox peaks. X-ray photoelectron spectroscopy (XPS) and high-resolution transmission electron microscopy (HRTEM) analyses of postcycled material confirm the structural and compositional stability of the material during cycling. This study advances the understanding of sulfur-rich materials in sodium-ion batteries across various electrolytes, particularly ionic liquids.

## Graphical abstract

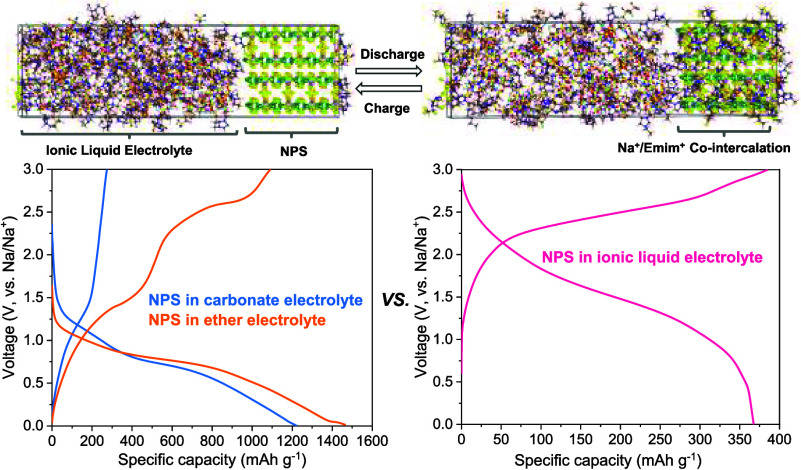

## Introduction

The demand for advanced battery technologies with high energy density and long cycle life has driven significant interest in lithium–sulfur (Li–S) batteries, which offer a theoretical capacity of 1672 mAh/g and are cost-effective and environmentally friendly. However, their practical application is hindered by issues such as the dissolution of polysulfides in traditional liquid electrolytes, leading to capacity fading and low Coulombic efficiency.^1,2^ Sodium-ion batteries (SIBs), utilizing abundant sodium resources, emerge as a promising alternative for lithium; yet, they also face similar challenges related to low energy density and the compatibility of existing liquid electrolytes toward sulfur electrode.^3^ A common strategy is to embed sulfur in high surface area carbon to form S/C composites and use transition metal compounds to enhance polysulfide conversion.^4,5^ However, polysulfides still diffuse to the lithium/sodium anode, where they form lower-order polysulfides and precipitate on the anode surface, causing corrosion and increasing impedance, which leads to capacity fading.

Recently, emerging sulfur-rich transition metal polysulfide (TMPS) cathodes, also known as sulfur-equivalent cathodes, such as W/MoS_*x*_,^6–9^ NbS_*x*_,^10^ TiS_*x*_,^11–13^ and Fe/Co/NiS_*x*_,^14,15^ (where *x* ranges from 3 to 7), possess higher electrical conductivity than insulating sulfur, resulting in superior kinetics during electrochemical reactions. Particularly, TMPS materials have demonstrated a high discharge plateau exceeding 1.5 V. For instance, MoS_2_ primarily contributes to discharge capacity below 0.6 V in lithium-ion batteries, and can only serve as an anode material.^16^ In contrast, MoS_3_ exhibits a dominant voltage plateau between 1.8 and 2.0 V vs Li/Li^+^ and delivers an initial capacity of 667 mAh/g, while also demonstrating a capacity of 460 mAh/g with a dominant voltage plateau between 1.2 and 1.8 V vs Na/Na^+^ as a sulfur-equivalent cathode material for Na–S batteries.^8^ For MoS_5_, the improved capacity reaches 902 mAh/g, with a more stable plateau between 1.8 and 2.0 V, contributing a higher proportion to the total capacity. Interestingly, excessive sulfur content leads to an additional plateau above 2.0 V, associated with the formation of liquid lithium polysulfides, mirroring the discharge behavior of lithium–sulfur batteries.^17^ The aforementioned studies used carbonate-based electrolytes, where MoS_3_ demonstrated better cycling stability than MoS_5_, potentially due to compatibility issues between high-sulfur TMPS materials and liquid electrolytes. Incorporating solid-state electrolytes can further address stability issues at the material interface. Yang et al.^7^ developed a sulfur-rich MoS_6_-based nanocomposite for lithium–sulfur batteries using a Li_7_P_3_S_11_ solid-state electrolyte, achieving an initial capacity of 1034 mAh/g between 1.0 and 3.0 V vs Li/Li^+^, and maintaining 550 mAh/g after 1000 cycles.

Ionic liquids (ILs) are recognized for their ability to create a protective coating on electrode surfaces during electrochemical reactions. This protective solid electrolyte interphase (SEI) plays a significant role in stabilizing the electrode and reducing side reactions, thereby improving battery longevity and performance.^18,19^ The research involving ILs in Li–S batteries attributes enhanced cycling performance primarily to the decreased solubility of polysulfides in these media, with a particular emphasis on the cathode side. This decrease in solubility is advantageous because it diminishes the polysulfide shuttling effect, a prevalent issue that contributes to capacity loss and reduced Coulombic efficiency in Li–S systems.^20^ To date, the specific roles of ILs in sulfur-rich TMPS materials have not been thoroughly investigated, but their effects may parallel the positive outcomes seen in lithium–sulfur batteries. Therefore, it is essential to conduct comprehensive studies to explore how ILs interact with sulfur-rich TMPS cathodes.

In this work, a phosphorus-containing sulfur-rich Nb_4_P_2_S_21_ (NPS) was employed as an electrode material in sodium-ion batteries, utilizing carbonate, ether, and IL-based electrolytes. To enhance both electronic conductivity and the reactivity of the material, Mo_2_CT_*x*_ MXene was blended with Nb_4_P_2_S_21_ to form a nanocomposite (MNPS) via ball milling. Cyclic voltammetry (CV) results indicated that NPS exhibited predominant redox couples below 1.5 V in the 0–3 V vs Na/Na^+^ range for both carbonate and ether-based electrolytes, with several complex redox couples observed in ether. Conversely, only a single pair of redox couples at 0.65/2.82 V was detected in the ionic liquid. Under a current density of 50 mA/g, the NPS material fully reacted with sodium in carbonate and ether electrolytes (Nb_4_P_2_S_21_ + 48Na → 4Nb + 2Na_3_P + 21Na_2_S), achieving an initial discharge capacity exceeding 1000 mAh/g, closing to its theoretical fully discharge capacity (1164 mAh/g). However, in the ionic liquid, the first discharge capacity of NPS was only 144 mAh/g, with 96.3% of this capacity originating from potentials above 0.8 V, indicating distinct characteristics of a cathode material. Interestingly, the sodiation discharge capacity below 0.8 V was notably suppressed, with no additional discharge plateau observed. The incorporation of conductive MXene into the MNPS composite resulted in a reduced polarization, yielding a redox couple at 1.22/2.66 V. After self-electrochemical activation, the material exhibited a maximum discharge capacity of 384 mAh/g, maintaining a median voltage above 1.5 V throughout 100 cycles. The ionic liquid electrolyte effectively suppresses sodiation of NPS at lower voltages, making it a promising cathode material for Na–S batteries.

## Experimental Section

### Synthesis of Nb_4_P_2_S_21_ (NPS)

Nb_4_P_2_S_21_ was synthesized using high-purity precursors, including niobium powder (99.999%, STREM, Germany), sulfur powder (99.999%, STREM, Germany), and red phosphorus (P) powder (99.999%, STREM, Germany). An excess of 1 atom % of phosphorus and sulfur was utilized relative to the stoichiometric requirements. The powders were combined with a KCl flux in a 2:1 ratio and subsequently sealed in a quartz ampule under high vacuum conditions using an oxygen/hydrogen welding torch. The ampule was then placed in a dual-zone furnace specifically designed for crystal growth. The synthesis process took place over 7 days, during which the reaction and formation of Nb_4_P_2_S_21_ occurred while maintaining a temperature gradient of 50 °C between the source zone (650 °C) and the growth zone (600 °C). After the heating phase, the sample was permitted to cool naturally to room temperature. Any excess halides were removed using distilled water, resulting in the formation of orange-brown Nb_4_P_2_S_21_ macrofibers.

### Synthesis of Nb_4_P_2_S_21_/Mo_2_CT_*x*_ (MNPS)

The synthesis of MNPS involved mixing 84% Nb_4_P_2_S_21_, 15% thin-layered Mo_2_CT*_x_*, and 1% MWCNTs, totalling 3 g. To this mixture, an additional 2 mL of acetonitrile (ACN) was added for better mixture and dispersion. The resulting mixture was sealed in a zirconia ball milling jar within an argon-filled glovebox and subject to planetary ball milling at 500 rpm for 6 h. Following this process, the final product was collected and vacuum-dried at 80 °C under light to obtain the MNPS composite.

### Apparatus

X-ray diffraction (XRD) was performed using a Bruker D8 ADVANCE X-ray powder diffractometer with Cu Kα radiation (Germany) to determine the crystalline structure of the samples. Field-emission scanning electron microscopy (SEM) was conducted with a Tescan MAIA3 XMH system (Czech Republic), equipped with energy-dispersive X-ray analysis (EDX) from Oxford Instruments, to observe the morphology and elemental composition of the as-prepared samples. Transmission electron microscopy (TEM) and high-resolution transmission electron microscopy (HRTEM) analyses were performed using a Zeiss LIBRA 200 MC Cs scanning TEM operating at 200 kV, also coupled with EDX from Oxford Instruments. The specific surface areas and pore size distribution were measured using a Brunauer–Emmett–Teller (BET) instrument (NOVAtouch 2200, USA) via nitrogen physisorption. Lastly, the surface composition of the samples was analyzed using X-ray photoelectron spectroscopy (XPS) with a SPECS spectrometer, equipped with a monochromatic Al Kα X-ray source (1486.7 eV) and a hemispherical electron analyzer (Phoibos 150). All XPS spectra are calibrated to C–C at ca. 284.5 eV, and fitted using the Shirley background subtraction method.

### Coin-Cell Battery Assembly and Measurements

A slurry containing Nb_4_P_2_S_21_ (NPS) or Nb_4_P_2_S_21_/Mo_2_CT_*x*_ (MNPS), carbon black, and poly­(vinylidene fluoride) (PVDF) was prepared in an argon-filled glovebox, achieving a homogeneous mixture in a weight ratio of 85:5:10 using *N*-methyl-2-pyrrolidinone (NMP) as the solvent. This slurry was then evenly coated onto aluminum foil and subjected to vacuum drying at 80 °C. Subsequently, electrodes with a diameter of 10 mm were produced, featuring an areal mass loading of 1.0 to 1.2 mg cm^–2^. These electrodes were assembled into CR2032 coin cells, with sodium foil serving as the counter electrode and a glass-fiber membrane as the separator. The electrolyte solutions employed were 1 M NaPF_6_ in a 1:1 volumetric mixture of ethylene carbonate (EC) and diethyl carbonate (DEC), 1 M sodium triflate (NaOTF) in triethylene glycol dimethyl ether (TREGDME), and 1 M sodium bis­(trifluoromethylsulfonyl)­imide (NaTFSI) in the ionic liquid 1-ethyl-3-methylimidazolium bis­(trifluoromethylsulfonyl)­imide ([Emim]­TFSI).

Electrochemical testing involved discharge and charge performance analysis conducted using a Neware battery test system (Neware BTS 8.0, Shenzhen, China). Additionally, cyclic voltammetry (CV) measurements of the coin cells were performed using an Autolab PGSTAT204 (Eco Chemie, Utrecht, Netherlands) with NOVA Version 2.1.7 software. Electrochemical impedance spectroscopy (EIS) and DRT analysis measurements were carried out at room temperature using an electrochemical workstation (Gmary Interface 1010 E, Warminster, USA).

### Equilibrium Simulation of NPS in NaTFSI/[Emim]­TFSI Electrolyte

Initial configurations were built using Packmol^21^ with 10 NaTFSI and 100 EMIMTFSI molecules, approximating realistic concentrations. These molecules were randomly placed above a fixed Nb_4_P_2_S_21_ slab to model a solid–liquid interface. The structure was converted to LAMMPS format using VMD and TopoTools.^22^ The force field parameters combined GAFF and OPLS-AA models.^23,24^ Na^+^, Emim^+^, and TFSI^–^ were parametrized using validated ionic liquid force fields, while Lennard-Jones parameters for the Nb_4_P_2_S_21_ slab were assigned based on chemically similar GAFF types. Bonded interactions were modeled with harmonic terms and OPLS-style dihedrals; nonbonded interactions used lj/cut/coul/long with a 10 Å cutoff and PPPM for long-range electrostatics.^25^ All electrolyte species were fully flexible. After energy minimization, molecular dynamics simulations were performed in LAMMPS with periodic boundary conditions, a 1 fs time step, and velocity-Verlet integration.^26^ The system was equilibrated at 1000 K for 2 ns (Langevin thermostat, 100 fs damping), annealed to 400 K over 1 ns, further equilibrated for 7 ns, and simulated at 400 K for 20 ns.

## Results and Discussion

The SEM images (Figure 1a,b) illustrate the distinct morphological differences between NPS and the nanocomposite MNPS. The SEM image of NPS shows a fibrous structure, characterized by its orange-brown coloration, indicating unique crystalline formation. The corresponding EDX spectrum (Figure S1) with a Nb/P/S ratio of 4.1:2.0:20.9 aligns with the experimental design. In the Mo_2_Ga_2_C MAX etched and exfoliated to produce Mo_2_CT_*x*_ MXenes, no significant MAX impurity peaks were observed (Figure S2a). The STEM image (Figure S2b) shows that the MXene used is predominantly composed of Mo_2_CT_*x*_ monolayer with a lateral size smaller than 100 nm. The ball-milled MNPS (Figure 1b) exhibits a more compact morphology consisting of primary small particles. This morphological modification is expected to enhance electronic conductivity and surface activity, potentially improving electrochemical performance. The presence of Mo in MNPS (Figure 1c) confirms the successful integration of Mo_2_CT_*x*_. The uniform distribution of all dominant elements suggests good homogeneity within the nanocomposite material. Further XRD pattern analysis (Figure 2d) comparing NPS and MNPS indicates that the ball milling process did not alter the structure of NPS in MNPS, and that the MXenes with 2θ peaking at ∼9.6° was successfully integrated with NPS. The nitrogen adsorption–desorption isotherms (Figure 1e) reveal surface area and porosity characteristics of NPS and MNPS. The BET analysis shows that the MNPS nanocomposite exhibits a significantly higher specific surface area of 21.3794 m^2^/g compared to 10.8792 m^2^/g for NPS. The pore size distribution, shown in the inset, indicates a higher pore volume for MNPS, facilitating enhanced ion diffusion and electrolyte accessibility. This increased surface area and porosity are likely due to the introduction of Mo_2_CT_*x*_ MXenes, contributing to improved electrochemical performance by enabling better electrode–electrolyte interactions and reaction kinetics.

**Figure 1:**
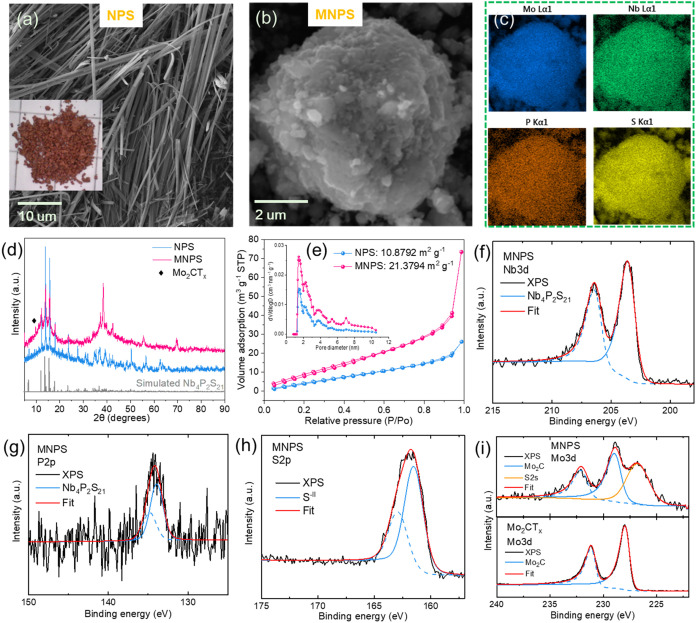
(a) SEM image and photograph of prepared NPS. (b) SEM image of MNPS and (c) the corresponding EDX-mapping of Mo, Nb, P and S elements. (d) XRD patterns and (e) nitrogen adsorption–desorption isotherms of NPS and MNPS (inset showing pore size distributions). High-resolution XPS spectra of (f) Nb 3d, (g) P 2p and (h) S 2p of MNPS. (i) Mo 3d XPS spectrum comparison of MNPS and Mo_2_CT_*x*_ MXene.

**Figure 2:**
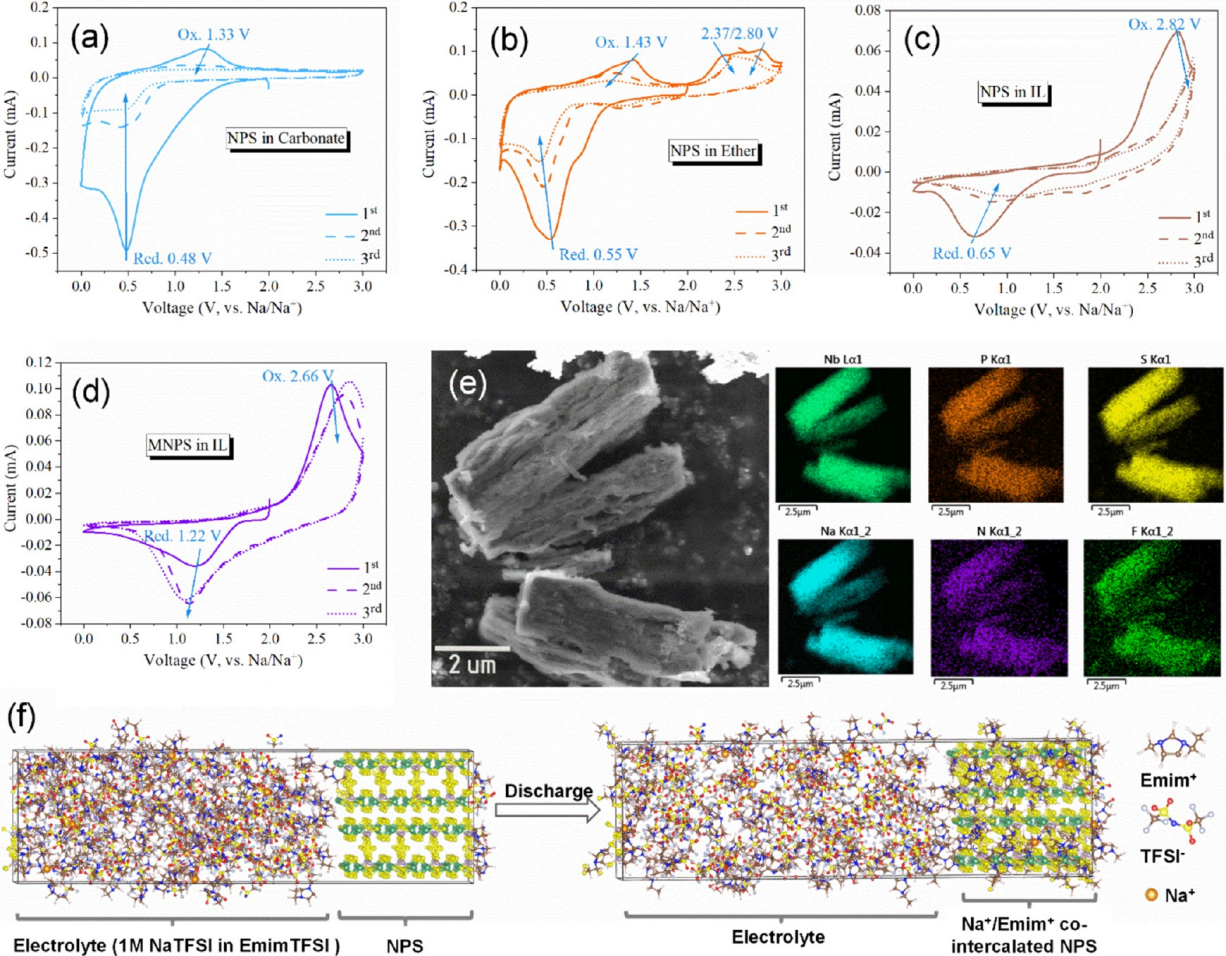
CV analysis of NPS at various electrolytes of (a) EC/DEC carbonate-based electrolyte, (b) triethylene glycol dimethyl ether (TREGDME), and (c) [Emim]­TFSI ionic liquid-based electrolyte, respectively. (d) CV curves of MNPS at ionic liquid-based electrolyte. Scan rate of CV: 0.1 mV s^–1^. Scan range: 0–3 V vs Na/Na^+^. (e) SEM and corresponding EDS mapping of negative scanned NPS from OCV to 0 V vs Na/Na^+^ [Emim]­TFSI ionic liquid-based electrolyte. (f) Schematic illustration of Na^+^ and Emim^+^ cointercalation in NPS during the charge process.

The chemical states of MNPS were determined using X-ray photoelectron spectroscopy (XPS). The survey spectra (Figure S3a) reveal the presence of key elements: Nb, P, S, and Mo. Figure 3f–h show the deconvoluted fitting of the Nb 3d, P 2p, and S 2p regions. The Nb 3d peaks at approximately 203.6 and 206.4 eV with a split spin–orbit component of 2.8 eV confirm the expected chemical environment. The P 2p deconvoluted peaks at 133.9 eV for P 2p_3/2_ and 134.8 eV for P 2p_1/2_ are attributed to P–S bonds within the PS_4_ units in transition metal thiophosphates.^27–29^ The S 2p peaks, appearing at 161.6 eV for S 2p_3/2_ and 163.0 eV for S 2p_1/2_, further corroborate this structure. The Mo 3d region (Figure 3i) in MNPS shows some overlap with the S 2p region, indicating the successful integration of NPS with Mo_2_CT_*x*_ to form the composite. Furthermore, a shift to higher binding energies in the Mo 3d peaks of MNPS compared to those in Mo_2_CT_*x*_ MXenes suggests electron transfer from Mo of MXene to the sulfur of NPS during contacting.^30,31^ Additionally, Figure S3b,e confirm the presence of carbide in the C 1s region, demonstrating the stability of MXenes within MNPS postball milling.

**Figure 3:**
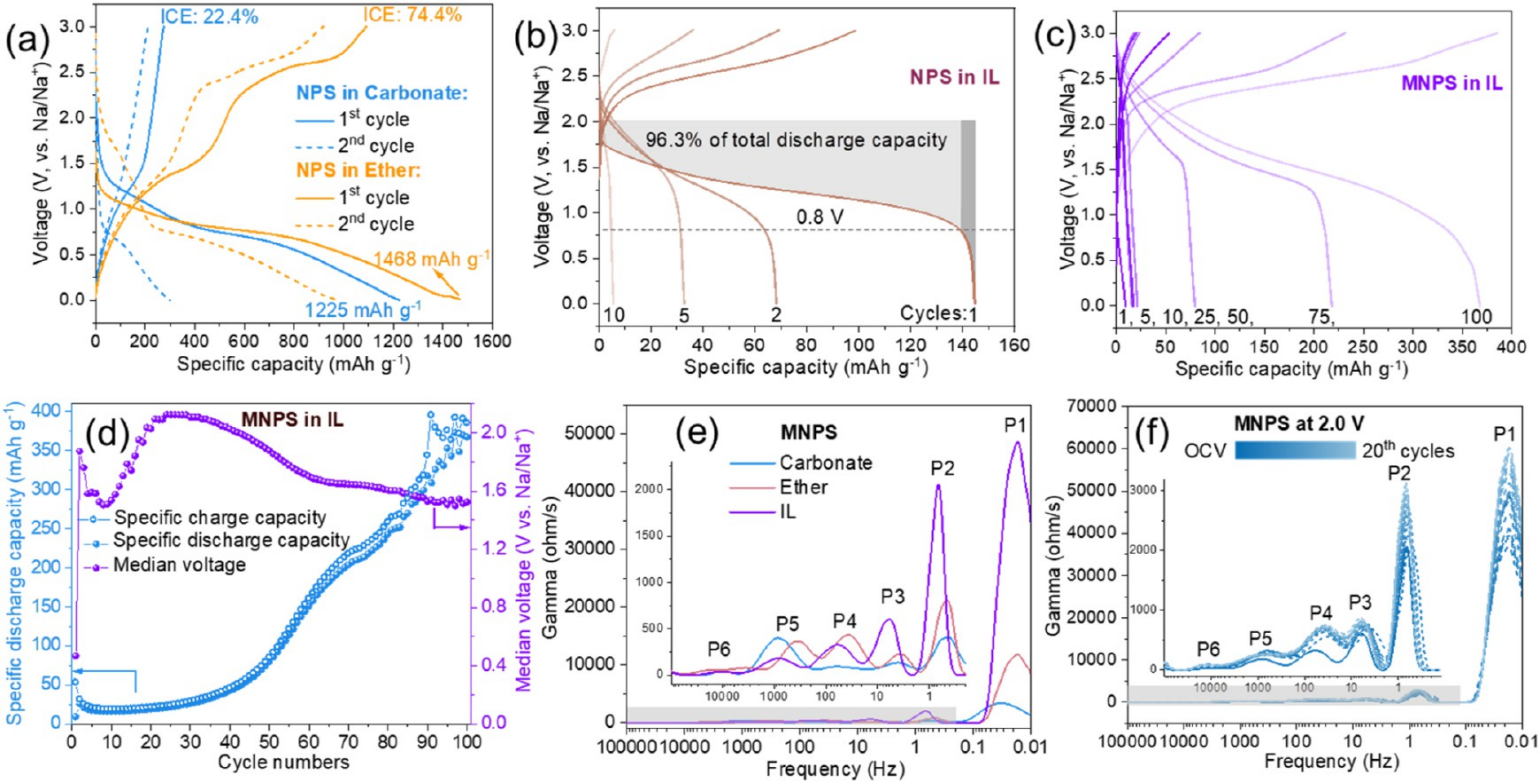
Galvanostatic charge–discharge of (a) NPS at carbonate (EC/DEC) and ether-based (TREGDME) electrolytes, (b) NPS at ionic liquid-based (EmimTFSI) electrolyte and (c) MNPS at ionic liquid (EmimTFSI) electrolyte. (d) Cycling stability of MNPS at the ionic liquid. Current density: 50 mA g^–1^. (e) DRT analysis of MNPS at various electrolytes. (f) DRT analysis of MNPS from OCP to 20^th^ cycles at 2.0 V.

Cyclic voltammetry results displayed in Figures 2 and S4 illustrate the redox behavior of Nb_4_P_2_S_21_ and Nb_4_P_2_S_21_/Mo_2_CT_*x*_ in various electrolyte systems, including carbonate (EC/DEC), ether (TREGDME), and ionic liquids ([Emim]­TFSI). The NPS electrodes in carbonate (Figure 2a) exhibit an oxidation peak at 1.33 V and a reduction peak at 0.48 V; however, the CV area quickly shrinks during subsequent cycles, with no significant oxidative peaks appearing, indicating a high degree of irreversibility in surface capacity. In the ether-based electrolyte (Figure 2b), NPS shows an oxidation peak at 1.43 V and a reduction peak at 0.55 V, as well as several additional oxidation peaks around 2.5 V. The peak area retention of NPS in ether is noticeably higher than that in carbonate, demonstrating more stable electrochemical performance. Similarly, the MNPS composite displays prominent redox peaks at low voltage ranges in both carbonate and ether electrolytes (Figure S4). Thanks to the integration of MXenes, the CV area retention for MNPS during cycling is significantly improved. In contrast, when NPS is tested in IL (Figure 2c), the CV curves reveal a single pair redox couple at 0.65/2.82 V, characterized by a large polarization voltage gap and low CV area retention in subsequent cycles. Notably, the integration of Mo_2_CT_*x*_ to form the MNPS composite results in a defined redox couple in the IL at a lower polarization gap of 1.22/2.66 V (Figure 2d). This shift indicates enhanced electrochemical kinetics and reduced polarization, highlighting the beneficial effects of Mo_2_CT_*x*_ on the overall performance of the composite material. Furthermore, this enhancement reinforces its potential as an effective cathode material for sodium-ion batteries.

To investigate the electrochemical reactions occurring in quasi-one-dimensional layered Nb_4_P_2_S_21_ within an ionic liquid environment, we conducted SEM and EDS analyses on the NPS electrode after the first discharge to 0 V under open-circuit voltage (OCV) conditions. As shown in Figure 2e, the material exhibits a fibrous, delaminated morphology, indicative of ion intercalation. Elemental mapping confirms the homogeneous distribution of Nb_4_P_2_S_21_ alongside Na, N, and F. The corresponding EDS spectrum in Figure S5 reveals an atomic ratio of Nb (from NPS): Na: N (from Emim^+^): F (from TFSI^–^) = 21.8:43.3:27.3:7.5, suggesting the cointercalation of Na^+^ and Emim^+^ during discharge. Figure 2f illustrates a schematic of cation intercalation at equilibrium under ionic liquid conditions. This experimental observation is consistent with previously reported Emim^+^ intercalation behaviors in layered two-dimensional materials such as MoS_2_ and graphite.^32–34^ However, due to the limited interlayer spacing and the relatively large ionic radius of Emim^+^, the reported capacity has typically remained below 50 mAh/g. The cointercalation of smaller Na^+^ ions offers a promising route to enhance the overall capacity. Many two-dimensional layered materials can accommodate both Emim^+^ and alkali metal ions due to their accessible interlayer spacing. Although Emim^+^ typically contributes a limited capacity because of its large ionic size, it can intercalate into certain layered hosts and induce interlayer expansion. Co-intercalation behavior has been reported in materials such as MoS_2_ and NiPS_3_; for example, NiPS_3_ forms Emim_0.41_Li_0.59_NiPS_3_ in a mixed-ion electrolyte.^35^ Given the structural similarity of Nb_4_P_2_S_21_ to these materials and the comparable ionic liquid environment, cointercalation of Na^+^ and Emim^+^ is likely in our system. To further examine the intercalation capability of Emim^+^ in NPS, we conducted a control experiment using a Na||NPS cell with neat EmimTFSI electrolyte (i.e., without any added sodium salt). As shown in Figure S9, an initial discharge process dominated by Emim^+^ intercalation is observed. With continued cycling, Na^+^ ions are gradually introduced into the electrolyte via stripping from the Na metal counter electrode, leading to a distinct voltage plateau at ∼0.15 V associated with subsequent Na^+^ intercalation.

Furthermore, we evaluated the constant current charge–discharge capabilities of the materials under different electrolyte conditions. Figure 3a shows the charge–discharge cycling curves of NPS in carbonate (EC/DEC) and ether-based (TREGDME) electrolytes. The initial discharge capacity of NPS in carbonate electrolyte was 1225 mAh/g, attributed to the complete electrochemical sodiation of Nb_4_P_2_S_21_ (Nb_4_P_2_S_21_ + 48Na → 4Nb + 2Na_3_P + 21Na_2_S), aligning with the theoretical capacity of 1163 mAh/g. However, the initial Coulombic efficiency (ICE) was only 22.4%. In contrast, the first discharge capacity of NPS in ether-based electrolyte reached 1468 mAh/g with an ICE of 74.4%. This result indicates that the sulfur-rich NPS behaves similarly to other sulfur-rich TTPS, exhibiting less stability in carbonate electrolytes compared to ether-based electrolytes.^17,36^ Figure 3b illustrates the charge–discharge profiles of NPS in IL. Interestingly, the voltage versus specific capacity curve demonstrates that 96.3% of the total initial discharge capacity (144 mAh/g, corresponds to the insertion of approximately 6 cations (Na^+^ and/or Emim^+^) per Nb_4_P_2_S_21_ formula unit) is concentrated above 0.8 V, indicating a strong performance in the higher voltage range. However, its capacity rapidly decreases within the first 10 cycles. The higher discharge capacity observed in Nb_4_P_2_S_21_, compared to previously reported values for 2D MoS_2_ and graphite with pure Emim^+^ intercalation, is attributed to the cointercalation of both Na^+^ and Emim^+^ ions.

Figure 3c illustrates the charge–discharge profiles of the MNPS composite in ILs over 100 cycles. The voltage vs specific capacity curves demonstrates a gradual increase in capacity, accompanied by a stable voltage plateau, indicating consistent electrochemical behavior throughout the cycling process. Notably, the MNPS composite exhibits a robust discharge capacity attributed to the integration of Mo_2_CT_*x*_ MXene, achieving significant values while maintaining high voltage levels. As the cycles progress from 1 to 100 (Figure 3d), the discharge capacity of MNPS gradually rises, reaching a maximum of 384 mAh/g, with a slight decrease observed after 97 cycles, corresponding to a specific energy of ∼ 576 Wh/kg. This value exceeds those of several mainstream sodium-ion cathode materials, such as Na_3_V_2_(PO_4_)_2_F_3_ (∼507 Wh/kg), NASICON-type Na_3_V_2_(PO_4_)_3_ (∼400 Wh/kg), and layered oxides (e.g., P2–Na_0.55_[Ni_0.1_Fe_0.1_Mn_0.8_]­O_2_, ∼350 Wh/kg).^37,38^ The corresponding dQ/dV analysis of the MNPS electrode reveals a small reduction peak around 0.58 V (Figure S11), which may be associated with the electrochemical activity of the MXene component. A gradual increase in capacity during the initial cycles suggests a self-activation process in the MNPS electrode when cycled in the IL electrolyte. This behavior is likely attributed to the initially poor wetting of the electrode by the viscous IL, as evidenced by the low first-cycle discharge capacity (Figure S12) compared to pristine NPS. With continued cycling, improved electrolyte infiltration, partial structural refinement of NPS particles, and interfacial stabilization may enhance ion accessibility and electrode kinetics. Additionally, partial adsorption of TFSI^–^ anions on the high-surface-area composite may contribute to the initially higher charge capacity. Importantly, the median voltage remains consistently above 1.5 V throughout the 100 cycles. This behavior underscores the effectiveness of MNPS as a cathode material in ILs, demonstrating its ability to deliver considerable discharge performance while minimizing contributions from lower voltage regions. In the ionic liquid environment, the material does not exhibit the extended Na^+^ reaction slope and plateau below 0.8 V as observed in carbonate and ether-based electrolytes. This difference may be attributed to the intercalation-induced passivation effect of the ionic liquid, which likely protects the material surface and suppresses further sodiation into the decomposition of high-capacity products such as Na_2_S, and Na_3_P. Monolayer Mo_2_CT_*x*_ shows a distinct discharge plateau at 0.4–0.6 V and an initial capacity over 2000 mAh/g (Figure S8a), which is significantly higher than that of other MXenes such as Ti_2_CT_*x*_ (∼360 mAh/g),^39^ likely due to Na^+^ deposition on its conductive surface. In contrast, this feature is absent in the MNPS composite, indicating that the electrochemical activity of MXenes is largely suppressed. This may result from surface coverage or close contact with NPS particles during ball milling (as depicted in Figure 1i), which limits interaction with the electrolyte. These results suggest that MXenes primarily serves as a conductive matrix, while the capacity mainly arises from the NPS phase.Moreover, we compared the normalized charge/discharge profiles (Figure S8b) of (i) MNPS after 100 cycles, (ii) NPS without MXenes after the first cycle, and (iii) MXenes alone after the first cycle. The profile of cycled MNPS closely matches that of NPS, and clearly differs from that of MXenes, indicating that the capacity after 100 cycles mainly originates from the Nb_4_P_2_S_21_ component rather than from MXenes. Notably, the average discharge voltage of MNPS gradually decreases from cycle 25 to 100, likely due to particle size reduction during cycling.^40^

To elucidate the behavior of the material in different electrolytes, we employed dynamic relaxation time (DRT) analysis. The DRT spectra provide a more detailed deconvolution of the electrochemical response processes within the material compared to impedance spectroscopy (EIS). Figure 3e illustrates the DRT analysis for MNPS across various electrolyte systems. The peak 1 (P1) in the low-frequency region is associated with the solid diffusion of cations through the bulk material, showing significantly higher resistance in ILs compared to carbonate and ether electrolytes. This increased resistance is attributed to the sluggish intercalation kinetics of the larger-sized Emim^+^ cations. Peaks P2 and P3 in the midlow frequency region are typically linked to charge transfer processes, and it is evident that MNPS exhibits higher charge transfer resistance in IL. Furthermore, several peaks in the midhigh frequency range suggest the presence of multiple interfaces and complex electrochemical processes occurring within the material. This observation of elevated charge transfer impedance and diffusion resistance of ions within the material in IL may limit the further sodiation of sodium ions at low voltages, thereby allowing the material to maintain high-capacity output only at higher voltages. Figure 3f displays the DRT analysis of MNPS from open circuit potential (OCP) through to the 20th cycle at 2.0 V. The peak shapes of the material show minimal changes, indicating stability throughout the cycling process. Only minor shifts in peak positions and variations in intensity are observed, reflecting alterations in the electrochemical kinetics during the electrochemical activation process. To further investigate the suppression of low-voltage capacity in the ionic liquid system, EIS measurements were conducted at three electrochemical states: open-circuit voltage (OCV, ∼2 V), after full discharge to 0 V, and after recharge to 3 V, as shown in Figure S10. At OCV, the semicircle is smallest and the Warburg region steep, indicating low charge-transfer resistance but limited ion diffusion, likely due to the dominance of Emim^+^ transport. Upon discharge to 0 V, the semicircle increases and the Warburg slope flattens, suggesting increased interfacial resistance—possibly from SEI formation—and enhanced Na^+^ diffusion as the electrode becomes electrochemically activated. Moreover, Emim^+^ cations, due to their large size and low mobility, may intercalate into the layered structure and sterically block Na^+^-accessible channels, particularly those required for conversion-type reactions. Unlike Na^+^, which can participate in both intercalation and deep conversion, Emim^+^ is generally limited to shallow intercalation and does not induce structural transformations. These effects together may restrict low-voltage sodiation pathways, shifting the reaction toward higher-potential processes.

We further characterized the MNPS composite after 100 cycles. The SEM image in Figure 4a reveals the morphology of MNPS, which is similar to the particulate texture observed before cycling in Figure 1b. This consistency indicates that the material retains its structural integrity even after extensive cycling. Figure 4b shows the uniform distribution of key elements—N, Mo, Nb, P, S, Na, and F—within the material postcycling, confirming effective integration and stability of the composite throughout the electrochemical process. Additionally, the XPS analysis further depicts the chemical states of the elements in the MNPS composite after cycling. Figure 4c displays the Nb 3d XPS spectrum of the postreacted MNPS, which presents two pairs of deconvoluted peaks. The peaks at Nb 3d_5/2_ at 206.8 eV and Nb 3d_3/2_ at 209.6 eV indicate the presence of higher oxidation state Nb­(V), likely resulting from oxidation during the charging or sample transfer processes. Additionally, another pair associated with NPS appears at Nb 3d_5/2_ at 204.5 eV and Nb 3d_3/2_ at 207.3 eV, which is approximately 0.9 eV higher than before cycling, which can be attributed to partial oxidation of the material during electrochemical cycling. The P 2p peaks (Figure 4d) at 133.2 and 134.1 eV are consistent with the oxidation states of phosphorus found in the PS_4_ units, similar to those observed before cycling. In other conventional thiophosphate anodes, phosphorus typically remains in the 0 to −3 oxidation states after cycling (e.g., P + Na → Na_3_P). This finding suggests that the PS_4_ units within NPS maintain stability throughout the cycling process. The S 2p spectrum (Figure 4e) reveals a pair of deconvoluted peaks at S 2p_3/2_ at 161.6 eV and S 2p_1/2_ at 163.0 eV, which are the same as those measured before cycling. Lastly, the presence of a single pair of doublets in the Mo 3d spectrum (Figure 4f) and the carbide peaks in the C 1s spectrum (Figure S6) further indicate that the MXene component remains stable during cycling.

**Figure 4:**
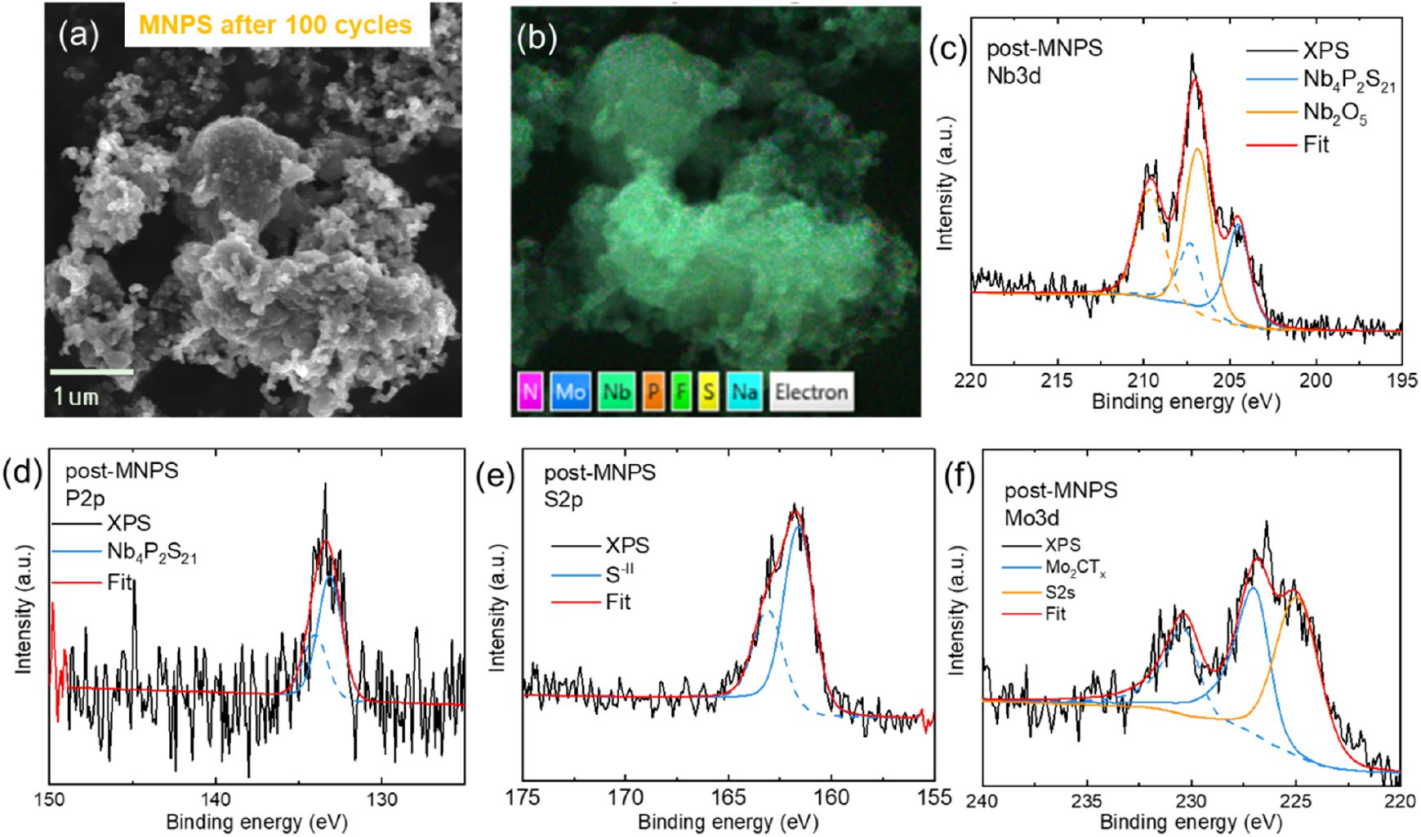
Analysis of postreacted MNPS over 100 cycles. (a) SEM and (b) EDX mapping. High-resolution XPS spectra of (c) Nb 3d, (d) P 2p, (e) S 2p, and (f) Mo 3d.

Further analysis using TEM and HRTEM were conducted to compare the microstructural changes of the material before and after cycling. The TEM image of postreacted MNPS over 100 cycles (Figure 5f) exhibits a less pronounced particulate texture compared to that observed before cycling (Figure 5a). This refinement in particle morphology may be a result of the repeated sodiation and desodiation processes, which modifies the material structure. The corresponding EDS mapping demonstrates a uniform distribution of elements, including Nb, P, S, and Mo, both before and after cycling, reinforcing the stability of the composite throughout the electrochemical reactions. Figure 5b presents an HRTEM image of the MNPS composite, highlighting the presence of thin-layered MXenes, indicated by red arrows, alongside Nb_4_P_2_S_21_. The measured interlayer spacing of approximately 5.61 Å aligns with the (310) crystal plane of NPS, as depicted in Figure 5c. Furthermore, analysis of other sample regions (Figure S7) revealed that NPS maintains its long-range structural integrity after ball-milling with MXenes. Figure 5d displays the XRD patterns of MNPS under various cycling conditions. Upon initial discharge to 0 V, the attenuation in diffraction intensity can be attributed to Na^+^ intercalation-induced structural distortion, while the primary diffraction patterns remain consistent with pristine NPS. Subsequent charge to 3 V resulted in significant recovery of diffraction intensity, with peak positions maintaining excellent correspondence with standard NPS patterns, indicating remarkable structural stability during Na^+^ extraction in ionic liquid electrolyte. Notably, after 100 cycles, the XRD pattern in the distinctive low-angle region (2θ ≈ 9.6°) exclusively displayed the MXene characteristic peak, with a complete disappearance of NPS signatures. The HRTEM analysis after 100 cycles (Figure 5e) confirmed the persistent presence of thin-layered MXenes. However, the NPS-related lattice fringes exhibited noticeable blurring, suggesting possible particle pulverization from repeated intercalation/deintercalation processes. Nevertheless, the well-preserved charge/discharge profiles throughout extended cycling, without the emergence of new electrochemical plateaus, strongly indicate that the fundamental electrochemical characteristics of Nb_4_P_2_S_21_ remain intact.

**Figure 5:**
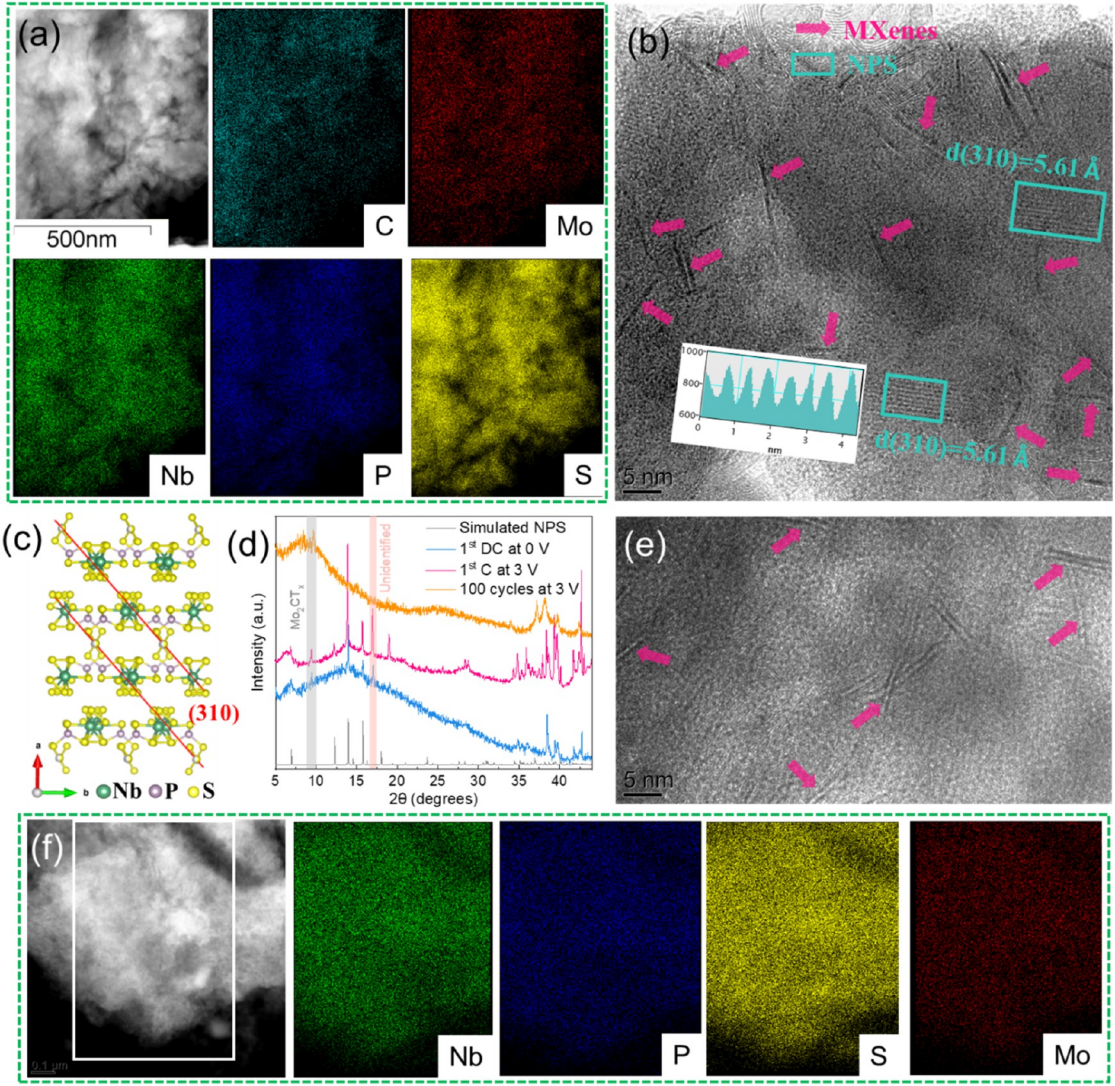
(a) TEM and EDX mapping and (b) HRTEM image of MNPS before cycling. (c) Structural illustration of NPS with crystal plane of (310). (d) XRD patterns of MNPS upon cycling. (e) HRTEM image and (f) TEM and EDX mapping of MNPS after 100 cycles.

It should be noted that although the NaTFSI–[Emim]­TFSI ionic liquid system was effective in modulating the electrochemical behavior of Nb_4_P_2_S_21_, its compatibility with metallic sodium is limited. As shown in our Na||Na CV tests (Figure S13), repeated cycling leads to a gradual decline in plating/stripping peak intensity, suggesting parasitic reactions between Na metal and IL. These observations are consistent with previous reports on the instability of imidazolium-based ILs with alkali metals.^41^ As shown in Figure S14, Na∥Na symmetric cells in organic electrolytes exhibit relatively stable stripping/plating behavior with moderate polarization at both 0.5 and 1 mA/cm^2^. In contrast, the cells cycled in the ionic liquid display an initial polarization that is tens of times higher, indicating sluggish Na kinetics and a markedly larger interfacial resistance, in agreement with the DRT analysis. Moreover, asymmetric stripping/plating capacities are observed during subsequent cycles, which can be attributed to preferential SEI formation on one electrode, hindering uniform Na deposition and dissolution. At an elevated current density of 1 mA/cm^2^, the electrodes fail to reach the preset capacity, suggesting that accelerated SEI growth under high current further restricts Na transport. Therefore, we acknowledge that this IL may not be suitable for long-term sodium–metal-based full cells. However, the objective of this work is not to develop a practical full-cell configuration but rather to demonstrate how the IL environment can suppress low-voltage conversion reactions and enable tunable intercalation behavior. The current half-cell configuration still provides meaningful mechanistic insights into voltage regulation, cation cointercalation, and interfacial effects. For practical application, future work will explore the use of more stable counter electrodes, such as presodiated hard carbon, Na alloys, or activated carbon, to avoid direct contact between metallic sodium and the IL.

## Conclusions

In this study, we synthesized a nanocomposite of Nb_4_P_2_S_21_ (NPS) integrated with Mo_2_CT_*x*_ MXene (MNPS) and evaluated its electrochemical properties in various electrolytes for sodium-ion batteries. In ionic liquid electrolytes, the system exhibits distinct electrochemical behavior compared to conventional carbonate and ether-based systems, particularly in terms of a higher average working voltage. This improvement is attributed to the cointercalation of Na^+^ and Emim^+^ into the NPS framework, resulting in a higher discharge capacity than previously reported EMIM^+^-only intercalation systems (e.g., MoS_2_ and graphite). Characterization techniques, including XRD, SEM, EDX, XPS, and HRTEM, confirmed the structural integrity and homogeneity of the composite. Cyclic voltammetry and galvanostatic charge/discharge tests in NaTFSI/[Emim]­TFSI electrolyte showed that NPS retains over 96.3% of its capacity above 0.8 V due to a cointercalation of Na^+^ and Emim^+^, while the incorporation of MXene in MNPS results in lower polarization and enhanced cycling stability, with a median voltage above 1.5 V over 100 cycles and a maximum discharge capacity of 384 mAh/g. DRT analysis suggests that the MNPS composite exhibits limited ion diffusion and charge transfer kinetics in ionic liquids due to the larger size of Emim^+^, which may also contribute to surface passivation and suppression of deep sodiation reactions. Further XPS, XRD, and HRTEM characterizations suggest that the material maintains structural stability upon cycling. Overall, these observations position sulfur-rich NPS as a viable candidate for cathodes in sodium-ion batteries within ionic liquid environments, paving the way for the development of sulfur-equivalent cathodes.

## Supplementary Material



## Data Availability

The data sets generated during and/or analyzed during the study are accessible via the Zenodo repository: 10.5281/zenodo.15065159.

## Notes

Details about EDS spectrum, XRD, STEM images, XPS, cyclic voltammograms, and HRTEM images (PDF)
