# Supplementary material for: Ionic Liquid Electrolyte Suppresses Deep Sodiation in Nb4P2S21/Mo2CT x Enabling Transition from Mixed-Voltage to Pure High-Voltage Operation for Sodium-Ion Battery Cathodes
Source: ACS Appl Mater Interfaces. 2025 Oct 1;17(41):57392–402. doi: 10.1021/acsami.5c10976 (PMC12532091; doi:10.1021/acsami.5c10976)
Supplement: Supplementary file 1 [file am5c10976_si_001.pdf]

Supporting Information

Ionic Liquid Electrolyte Suppresses Deep Sodiation in $Nb_4P_2S_{21}/Mo_2CT_x$ Enabling Transition from Mixed-Voltage to Pure High-Voltage Operation for Sodium-Ion Battery Cathodes

Heng Li[1,#], Lei Zheng[1,#], Zhongquan Liao[2], Vlastimil Mazánek[1], Qiliang Wei[3], Tomáš Hartman[1], Saeed Ashtiani[1], Bing Wu[1,], Zdenek Sofer[1,*]*

[1] Department of Inorganic Chemistry, University of Chemistry and Technology Prague, Technická 5, 166 28 Prague, Czech Republic

[2] Fraunhofer Institute for Ceramic Technologies and Systems (IKTS), Maria-Reiche-Strasse 2, 01109 Dresden, Germany

[3] Institute of Micro/Nano Materials and Devices, Ningbo University of Technology, Ningbo, 315211, P.R. China

[#] H. L. and L. Z. contributed equally as the first authors.

* Corresponding Author. E-mail: wui@vscht.cz and zdenek.sofer@vscht.cz

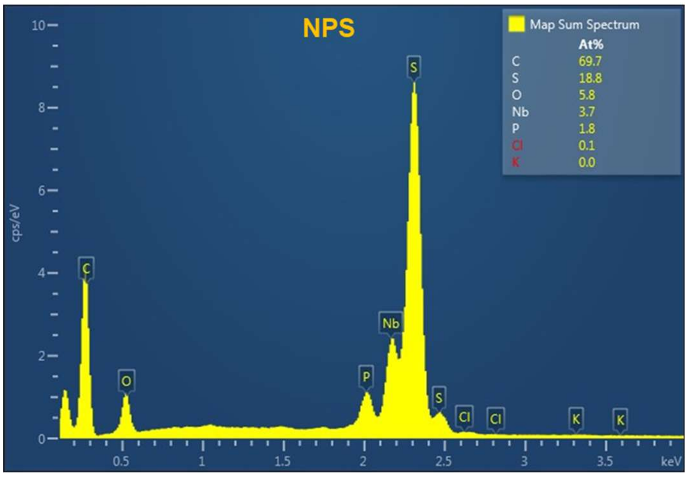

Figure S1 EDX spectrum of NPS.

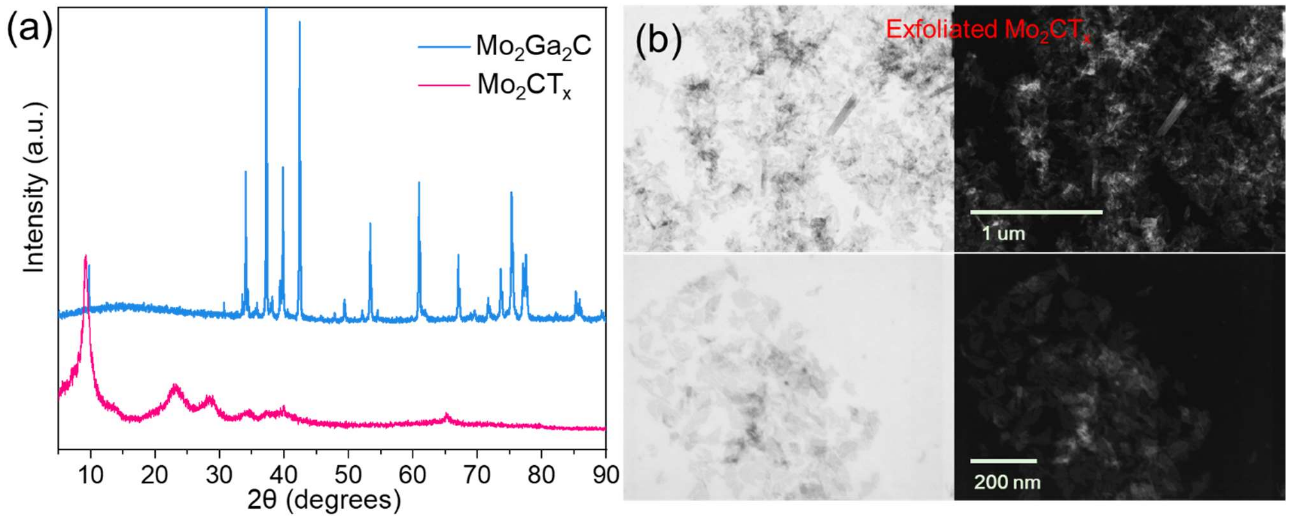

Figure S2 (a) XRD patterns of MAX Mo_2Ga_2C and MXene Mo_2CT_x. (b) STEM images of exfoliated Mo_2CT_x at different magnifications.

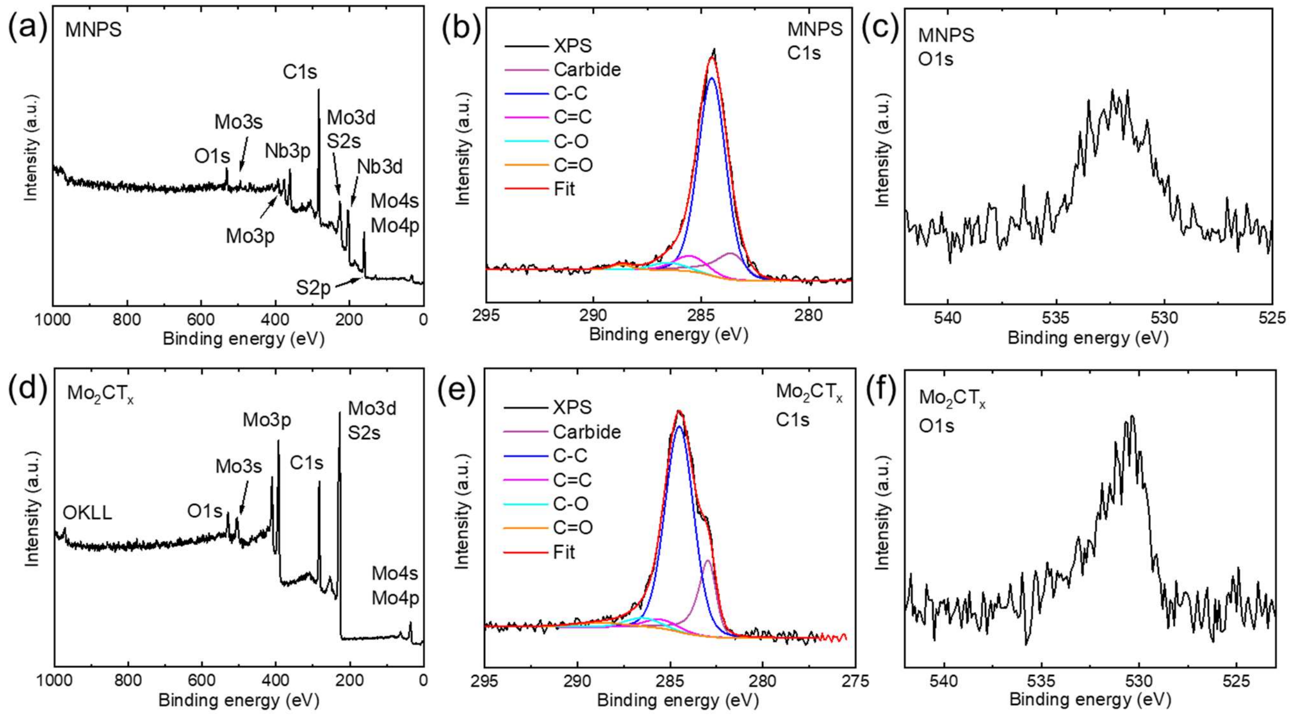

Figure S3 (a) XPS survey of MNPS and its high-resolution spectra of (b) C 1s and (c) O 1s. (d) XPS survey of Mo_2CTx and its high-resolution spectra of (e) C 1s and (f) O 1s.

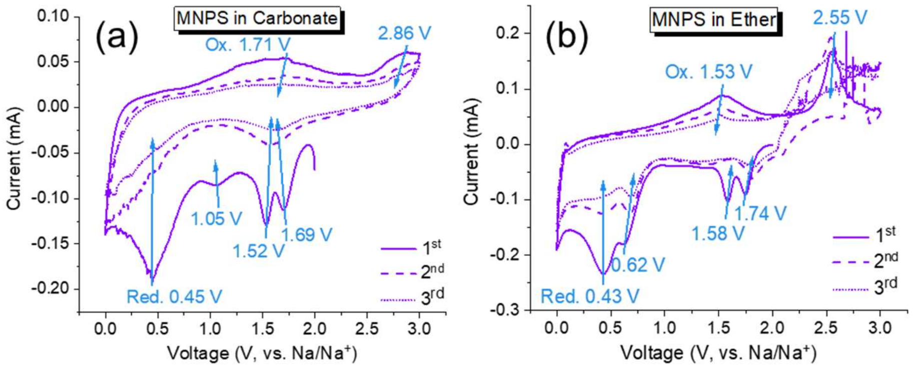

Figure S4 CV curves of MNPS at (a) carbonate-based and (b) ether-based electrolytes, respectively.

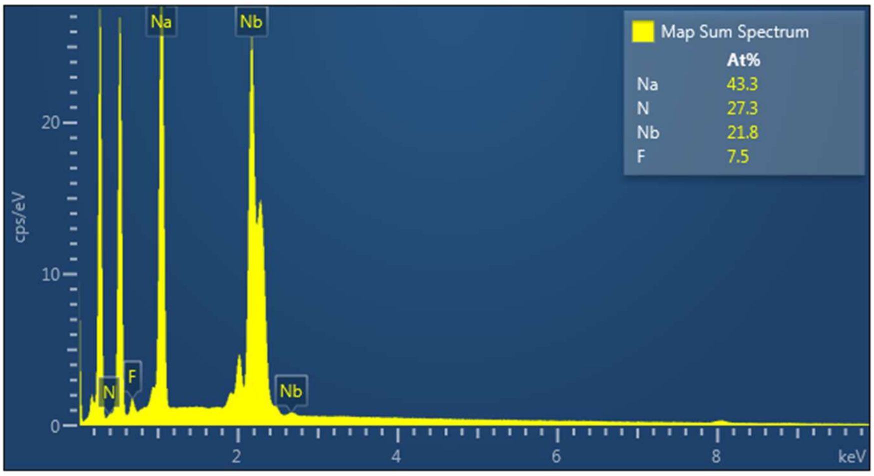

Figure S5 EDS spectrum of negative scanned NPS from OCV to 0 V vs. Na/Na+ [Emim]TFSI ionic liquid-based electrolyte.

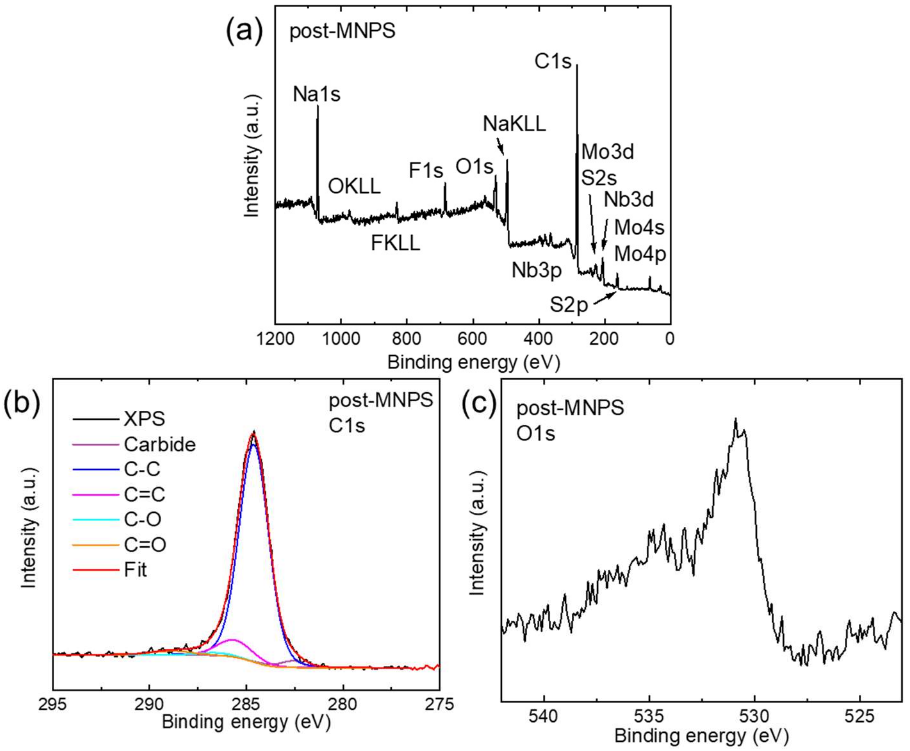

Figure S6 XPS of post-cycled MNPS. (a) Total survey, (b) C 1s and (c) O 1s spectra, respectively.

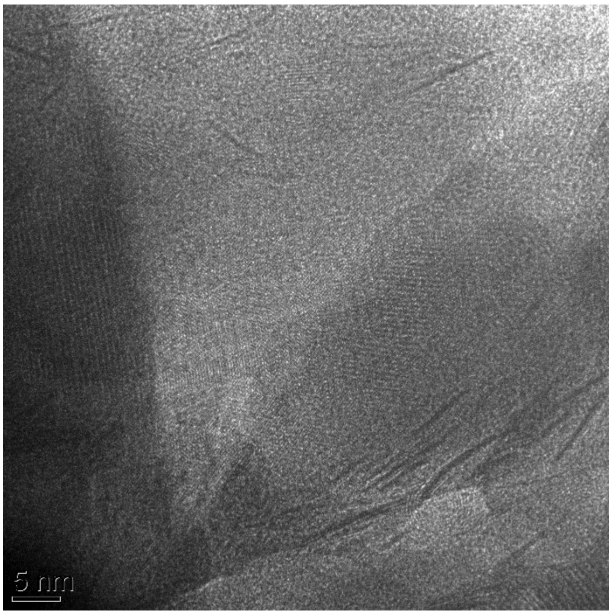

Figure S7 HRTEM image of another selected region in the MNPS composite.

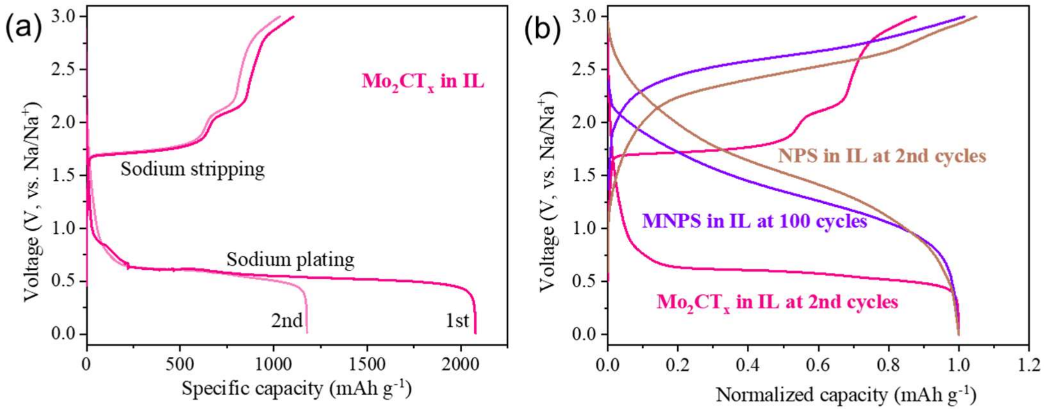

Figure S8 (a) Galvanostatic charge–discharge profile of monolayered Mo_2CT_x MXene in the ionic liquid electrolyte. (b) Normalized charge–discharge curves of MNPS after 100 cycles, pristine NPS electrode (without MXene) after the first cycle, and MXene after the first cycle.

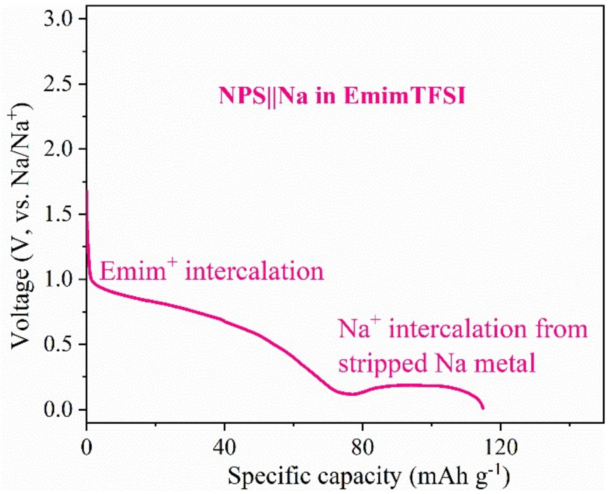

Figure S9 Initial discharge curve of NPS||Na cell with EmimTFSI electrolyte.

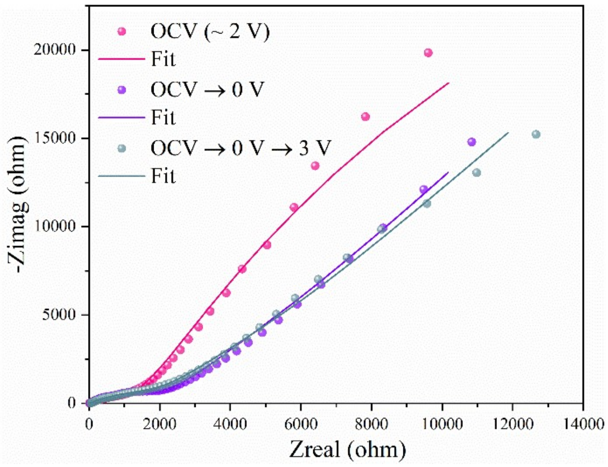

Figure S10 Nyquist plots of MNPS at different cut-off voltage.

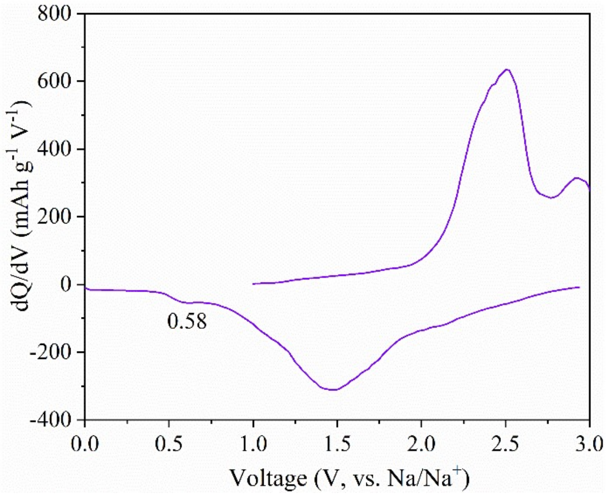

Figure S11 dQ/dV analysis of MNPS electrode at cycling of 384 mAh/g.

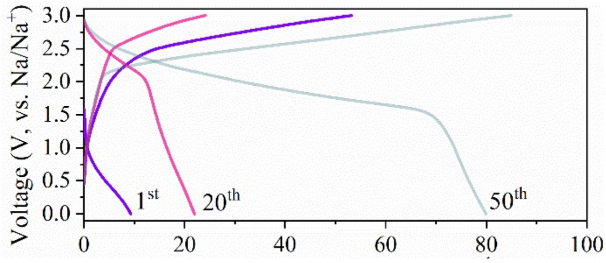

Figure S12 Charge-discharge curve of MNPS at 1st, 20th, and 50th cycles.

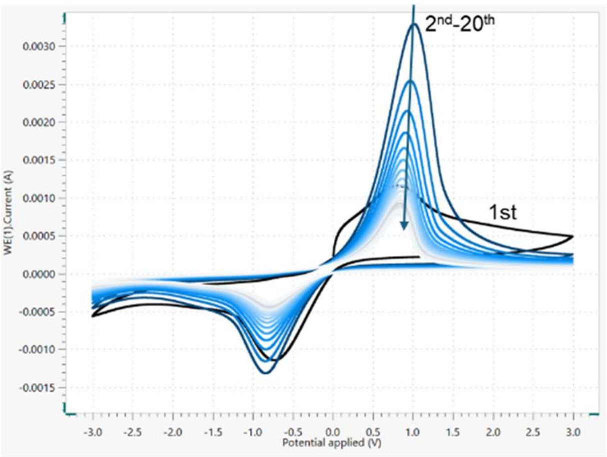

Figure S13 CV curves of Na||Na symmetrical cell with NaTFSI–[Emim]TFSI electrolyte.

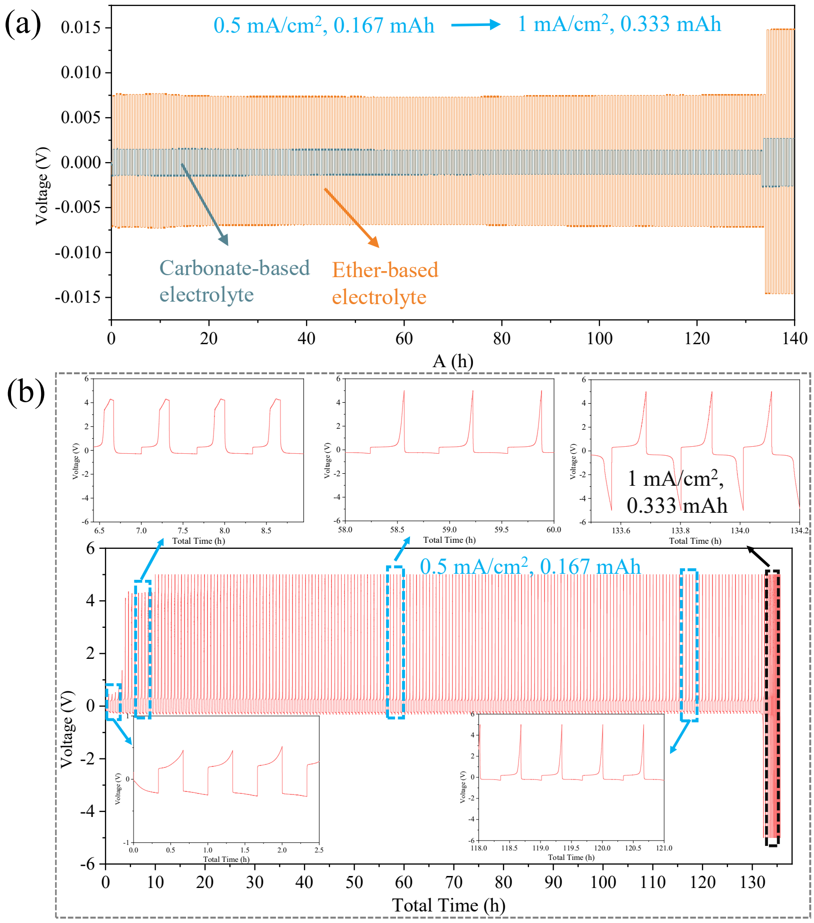

Figure S14 (a) Stripping/plating performance of Na||Na symmetric cells in carbonate- and ether-based electrolytes. (b) Stripping/plating performance of Na||Na symmetric cell with NaTFSI–[Emim]TFSI electrolyte, and corresponding voltage profiles with enlarged views highlighting representative cycling behaviors. All tests were conducted at 0.5 mA/cm^2 (0.167 mAh, 200 cycles) followed by 1 mA/cm^2 (0.333 mAh, 20 cycles).
